# Evidence for feminized genetic males in a flea beetle using newly identified X‐linked markers

**DOI:** 10.1002/ece3.70123

**Published:** 2024-08-12

**Authors:** Kim Rohlfing, Malte Grewoldt, Mathilde Cordellier, Susanne Dobler

**Affiliations:** ^1^ Institute of Animal Cell and Systems Biology, Universität Hamburg Hamburg Germany; ^2^ Present address: Department of Molecular Biology and Genetics Aarhus University Aarhus Denmark; ^3^ Present address: Institut für Biowissenschaften, Genetik – Populationsgenetik, Universität Rostock Rostock Germany

**Keywords:** *Altica lythri*, Chrysomelidae, female bias, feminization, genetic sex determination, *Wolbachia*, X‐linked marker

## Abstract

The equilibrium of sex ratios in sexually reproducing species is often disrupted by various environmental and genetic factors, including endosymbionts like *Wolbachia*. In this study, we explore the highly female‐biased sex ratio observed in the flea beetle, *Altica lythri*, and its underlying mechanisms. Ancient hybridization events between *Altica* species have led to mitochondrial DNA introgression, resulting in distinct mitochondrial haplotypes that go along with different *Wolbachia* infections (HT1‐wLytA1, HT1*‐ uninfected, HT2‐wLytA2, and HT3‐wLytB). Notably, beetles with some haplotypes exclusively produce female offspring, suggesting potential *Wolbachia*‐induced phenomena such as feminization of genetic males. However, the observed female bias could also be a consequence of the ancient hybridization resulting in nuclear–cytoplasmic conflicts between introgressed mtDNA and nuclear genes. Through transcriptomic analysis and the program SEX‐DETector, we established markers for genotypic sex differentiation for *A. lythri*, enabling genetic sexing via qPCR. Our findings suggest that feminization of genetic males is contributing to the skewed sex ratios, highlighting the intricate dynamics of sex determination and reproductive strategies in this flea beetle. This study provides valuable insights into the dynamics of genetic conflicts, endosymbionts, and sex ratios, revealing the novel phenomenon of genetic male feminization in the flea beetle *A. lythri*.

## Graphical abstract

This study investigates the highly female‐biased sex ratio in the flea beetle *Altica lythri*, examining potential mechanisms such as Wolbachia‐induced feminization of genetic males. Through transcriptomic analysis and the establishment of genotypic sex differentiation markers using the SEX‐DETector approach, we provide evidence that feminization of genetic males contributes to the skewed sex ratios in *A. lythri*.

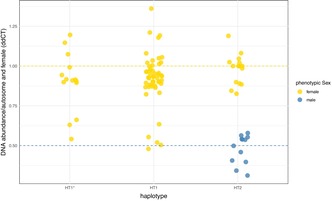

## 1 INTRODUCTION

When males and females have equal reproductive success, Fisher's (1930) principle posits a balanced sex ratio of 1:1 (Fisher, 1930). Yet, exceptions are common in nature due to various environmental and genetic factors such as parental investment, mate choice, genetic determinants of sex, hybridization, as well as endosymbionts (Albert & Otto, 2005; Courret et al., 2019; Hardy, 2002; Lindholm et al., 2016; Runemark et al., 2018; Trivers, 1972; West & Sheldon, 2002; Wood & Newton, 1991).

In hybrids especially, genetic conflicts can contribute to unbalanced sex ratios in populations. These conflicts may arise due to the mismatch between genetic elements from different parental species, leading to disruptions in reproductive processes, including sex determination and sex chromosome inheritance. Even conflicts between maternally transmitted genetic elements (such as mtDNA or endosymbionts) and the nuclear genome after ancient hybridization events can result in cytonuclear incompatibilities, potentially leading to disruptions in sex ratios (Perlman et al., 2015; Runemark et al., 2018).

The cytoplasmic alpha‐proteobacterium *Wolbachia*, is an endosymbiont found in almost two‐thirds of all invertebrates and notorious for causing female‐biased sex ratios (Werren et al., 2008; Werren & Windsor, 2000). As it is primarily transmitted through females (Werren et al., 2008), *Wolbachia* is often found to enhance its transmission by manipulating the reproductive biology of its hosts (Cordaux et al., 2011; Werren, 1997), for example, by inducing parthenogenesis, feminization of genetic males (Bouchon et al., 2008), or male killing (Hurst et al., 1999). It is thus affecting the dynamics of host populations (Cordaux et al., 2011; Werren et al., 2008).

Most populations of our study system, the flea beetle *Altica lythri*, exhibit a strongly female‐biased sex ratio. Furthermore, this beetle shows intriguing reproductive anomalies that may be due to ancient interspecific hybridization and infections by *Wolbachia* bacteria (Jäckel, 2011; Jäckel et al., 2013; Rohlfing et al., 2023). Interspecific hybridization between *Altica* species and subsequent backcrossing led to mitochondrial DNA introgression, resulting in three distinct haplotypes (HT1, HT2, and HT3) (Jäckel, 2011; Jäckel et al., 2013). *A. lythri* are mostly infected by *Wolbachia* bacteria and typically carry different strains (wLytA1, wLytA2, or wLytB) depending on their mitochondrial haplotype (HT1‐wLytA1, HT2‐wLytA2, and HT3‐wLytB). The haplotype HT1*, a slight sequence variant of HT1, was predominantly found to be uninfected by *Wolbachia*. Remarkably and regardless of the presence of a *Wolbachia* infection, beetles with HT1 or HT1* haplotypes exclusively produce female offspring. Those with HT2 or HT3 haplotypes exhibit a more or less balanced sex ratio in their progeny (Jäckel et al., 2013; Rohlfing et al., 2023). The reasons for the exclusively female offspring in HT1 and HT1* of *A. lythri* remain intriguing and are further investigated in this study.

One hypothesis to test is whether the female bias in HT1 is due to the infection with wLytA1, which could induce male killing, feminization of genetic males, or parthenogenesis. Even in the absence of an active *Wolbachia* infection, as observed in HT1*, this possibility persists if *Wolbachia* genes have been integrated into the nuclear genome. Horizontal gene transfer between *Wolbachia* and host genomes is known to occur, allowing the expression of *Wolbachia* genes that can have phenotypic effects including feminization (Asgharian et al., 2014; Leclercq et al., 2016; Woolfit et al., 2008). Alternatively, the female excess could be attributed to nuclear–cytoplasmic conflicts due to hybridization, thus resulting in the absence of males with HT1 or HT1* mtDNA. In extreme cases, nuclear–cytoplasmic conflicts driven by mitochondrial genes may result in the loss of the male sex, although this has so far only been demonstrated in hermaphroditic plants and snails (David et al., 2022; Fujii et al., 2011; Touzet & Budar, 2004).

Laboratory rearing of *A. lythri* HT1 females has shown that eggs laid by virgin females never hatch, thus allowing us to rule out true parthenogenesis in this system. Even females of the HT1/HT1* haplotype must mate with males of the HT2 and HT3 haplotypes to produce offspring (Jäckel, 2011; Rohlfing et al., 2023; Sanken, 2023). Paternity analyses supported that HT1 females actually reproduce by gynogenesis as hardly any paternal SNPs were detected in the offspring (Sanken, 2023). In this mode of reproduction, copulation is only necessary to trigger the development of the egg without contributing paternal genetic material to the offspring. In addition, *Wolbachia*‐induced male killing could be ruled out as a possible reason for the female bias in HT1/HT1*, as even in the earliest stages of egg development, no phenotypic males were detectable when using the phenotypic sex marker doublesex (Rohlfing et al., 2023).

In the present study, we investigate whether the alternative mechanism through which *Wolbachia* can manipulate sex ratios, namely feminization, could be responsible for the female excess in *A. lythri*. Reliable methods for genetic sex determination are crucial for answering this question. The identification of genetic sex in organisms can be achieved through cytogenetic methods (Pan et al., 2015), progeny cross‐testing experiments (Piferrer, 2001), or the identification of sex‐specific molecular markers (Devlin et al., 2001; Hawthorne, 2001; Ou et al., 2017). To date, no chromosomal markers exist for the genetic sex differentiation in *A. lythri*. In addition, although *A. lythri* females are XX and males XY, the sex chromosomes are homomorphic, making it impossible to assign a sex by karyology (Petitpierre et al., 1988).

Therefore, we utilized the SEX‐DETector approach (Muyle et al., 2016), where sex‐specific allele segregation patterns are analyzed in offspring of known pedigree, to establish genotypic sex differentiation markers for *A. lythri*. Using these markers, we demonstrate that genetic sexing of *A. lythri* larvae and adult beetles is possible by qPCR; copy number variation (CNV) of X‐linked genes (present in one copy in males, and two in females) is calculated relative to an autosomal gene (with two copies in both sexes). After evaluating the reliability of the genetic sexing procedure with the HT2 individuals, it was applied to test whether genetic males are hidden among the phenotypic females of HT1 or HT1*. Our results provide evidence that feminization of genetic males contributes to the occurrence of phenotypically all female offspring in HT1 and HT1*. This investigation sheds light on the intricate mechanisms underlying the dynamics of sex determination in *A. lythri*, revealing feminization of genetic males in a beetle species for the first time.

## 2 MATERIALS AND METHODS

### 2.1 Bioinformatic identification of sex chromosomal‐linked markers

#### 2.1.1 RNA extraction

A female beetle (collected in Büchen (53.479N, 10.632E), Germany) and a male beetle (collected in Wedel (53.581N, 9.693E), Germany) with haplotype HT2 were both collected in early spring 2019 and reared in a climate chamber at 17°C and a 12 h light/dark cycle. The virgin status of the female was confirmed by checking (at least) three egg clutches to make sure that no larvae were developing. The beetles were allowed to mate and their offspring were raised to the adult stage, totaling six male and four female offspring. Sex was determined morphologically based on the internal sex organs. Total RNA was obtained from these 12 samples by crushing the nitrogen‐frozen samples with a tube pestle and extracting them using the RNeasy Mini Plus kit (Qiagen, Hilden, Germany) according to manufacturer's instructions. Samples were sent for library preparation and sequencing to Novogene (UK) with Illumina paired‐end and a read length of 150.

#### 2.1.2 Read quality control and trimming

The overall read quality was assessed with FastQC (Galaxy Version 0.72; Andrews, 2015) and MultiQC (Galaxy Version 1.7; Ewels et al., 2016). An initial clipping of Illumina adapters was already performed by the sequencing company. Trimmomatic (Galaxy Version 0.36.6; Bolger et al., 2014) was used to process and filter reads as follows. The reads were trimmed at the 5′ and 3′ ends, removing bases with a Phred score of ≤20 by means of the Trimmomatic Trailing function (TRAILING:20). The reads were further refined using the sliding window function to remove fragments of 40 bases that had an average Phred score of ≤30 (SLIDINGWINDOW:40:30). Eventually, reads that had a length below 120 bp after processing were dropped (MINLEN:120).

#### 2.1.3 Reference assembly

To build a reference transcriptome assembly, RNA was obtained as described above from one HT1 female (caught in June 2018) and one HT2 male beetle (caught in April 2017). Samples were sent for library preparation and sequencing to Starseq (UK) with Illumina paired‐end reads with a 150 bp read length on a Nextseq500. The reads for the reference assembly were processed as follows on the high‐performance computing cluster “Hummel” at Universität Hamburg. Trimmomatic, implemented in Trinity 2.4.0 (Grabherr et al., 2011; Haas et al., 2013), was used with “ILLUMINACLIP:2:30:10 SLIDINGWINDOW:4:5 LEADING:5 TRAILING:5 MINLEN:25” options to trim and filter the reads. The processed and filtered reads from both samples were pooled and assembled de novo with Trinity (Galaxy Version 2.8.4; Grabherr et al., 2011; Haas et al., 2013) as paired data, with in silico normalization of reads and with a minimum contig length of 200 bp.

#### 2.1.4 Transcriptome quality assessment

The initial quality and completeness of the de novo transcriptome assembly were assessed with the Benchmarking set of the Universal Single‐Copy Orthologs program (BUSCO) options: ‐m transcriptome; version 3.0.2 (Simão et al., 2015), with the Arthropod lineage BUSCO set from OrthoDB; version 9 (Waterhouse et al., 2019).

To reduce redundancy of transcripts in the assembly (i.e., removing duplicates, isoforms, or fragmented transcripts), the tr2aacds script from the EvidentialGene package (‐log ‐mrnaseq; version Jan 2019, Gilbert, 2016) was used on the Trinity.genes file. The EvidentialGene output category “okay‐alt” set of transcripts which contained primary transcripts with high coding potential was used in further downstream analyses. Once again, the quality of de novo transcriptome assembly was examined with BUSCO and we confirmed that the number of duplicates in the assembly had been reduced through the EvidentialGene tr2cdsaa script. The trimmed paired‐end RNA reads of all 12 individuals were mapped separately to the transcriptome assembly. This was achieved using the Burrows‐Wheeler Aligner (BWA‐MEM, options: ‐a auto, paired_collection, analysis mode: Illumina; Galaxy Version 0.7.17.1; Li & Durbin, 2009) to control whether the transcriptome was representative and as preparation for further analyses. The statistics of the mapping were assessed with the default Stats function and the BedCov program of the SAMTools package (Galaxy Version 2.0.1; Li, 2011).

#### 2.1.5 Read filtering and variant calling

BAM files from all 12 individuals from the BWA‐MEM mapping were filtered with Samtools View (Galaxy Version 2.0.1; Li, 2011) to obtain only reliably mapped reads. Only those reads that had a mapping quality score (MAPQ) of ≥30 and that were mapped in a proper pair were retained, while unmapped reads were dropped. The filtered bam files were then sorted using Samtools Sort (Galaxy Version 2.0.1; Li, 2011) to ensure that the respective files were structured in the same order and to accelerate the following analyses.

To detect single nucleotide polymorphisms (SNPs) in the 12 *A. lythri* individuals, parents, and offspring alike, the genotyping tool reads2snp (version 2.0; Tsagkogeorga et al., 2012) was used. The minimum number of reads to be taken into account for SNP calling was set to 3 (option ‐min 3). By default, reads2snp removes paralogous sequences with the Paraclean program. However, this has been disabled (option ‐par 0) because gene copies on allosomes may resemble paralogs. To keep the contigs in the output alr‐ and gen‐file in the same order, the number of threads was set to 1 (option ‐nbth 1). In addition, for accurate SNP calling, the base quality was required to be greater than 20 and the mapping quality to be greater than 10 (options ‐bqt 20; ‐rqt 10). The reference transcriptome of *A. lythri*, as well as sorted bam files of all 12 individuals, were provided as input files. The produced alr‐ and gen‐files were used for the subsequent SEX‐DETector analyses.

#### 2.1.6 SEX‐DETector

In preparation for the main SEX‐DETector analysis and as a prerequisite for data from a cross, the perl script SEX‐DETector_prepare_file.pl from the SEX‐DETector package was used. This step summarizes the results obtained in the reads2snp genotyping by producing an alr_gen_summary output file that only shows one occurrence of each possible SNP in the dataset as well as its quantity, thus enabling SEX‐DETector to run faster.

For the main SEX‐DETector analysis, the input files used were the alr_gen‐ and alr‐file (options: ‐alr_gen; −alr, optional) from the reads2snp analysis together with the alr_gen_summary‐file (‐alr_gen_sum) from the SEX‐DETector preparation. In addition to listing hetero‐ or homogametic parents (‐hom_par; ‐het_par) and offspring (‐hom; ‐het), a XY system was specified (‐system xy), which was only relevant for the naming of the output files. The sex chromosome system (SCS) was detected automatically by the program independently of this option. SEX‐DETector assigned the transcriptome's contigs to the categories “sex‐linked,” “autosomal,” or “lack‐information” according to their calculated posterior probabilities. The contigs from the categories “sex‐linked” and “autosomal‐linked” were used to design primers for qPCR (see below).

### 2.2 Evaluation of X‐linked markers using qPCR

#### 2.2.1 Extraction of genomic DNA

DNA from adult *A. lythri* beetles was extracted by crushing nitrogen‐frozen individuals with a tube pestle and extracting using the DNeasy Blood and Tissue Kit (Qiagen, Hilden, Germany) according to manufacturer's instructions. The phenotypic sex of the adults was determined morphologically based on the internal sex organs. The sex of the larvae cannot be distinguished phenotypically and was therefore determined by RT‐PCR of the splice variants of doublesex, as previously reported by Rohlfing et al. (2023). To do so, frozen larvae were divided in half, with one half used for phenotypic sexing via RNA and the other for genotypic sexing with DNA extraction. RNA was extracted with the MN RNA XS Kit (Macherey–Nagel, Düren, Germany), reverse transcribed with Superscript III and then used for amplification of the phenotypic marker doublesex for phenotypic sexing (Rohlfing et al., 2023). The DNA was extracted with the tissue extraction protocol of the forensic kit (Analytik Jena, Jena, Germany) according to manufacturer's instructions. The mtDNA haplotype was determined via PCR‐RFLP as described in a previous study (Jäckel, 2011).

#### 2.2.2 Primer design for candidate marker loci

Beyond their assignment to the groups “sex‐linked” and “autosomal‐linked,” further information was used to select the target sequences for qPCR primer design. Selected sequences required to have a hit of 80% sequence identity and display an e‐value of 1e‐20 in tblastx searches on linkage group X (LGX) in *Tribolium castaneum* and linkage group 10 (LG10) in *Popillia japonica*, the latter being the putative X chromosome of *P. japonica*. The same filters were set for autosomal target sequences; however, they were required to have a hit on any chromosome but on LGX in *T. castaneum* and LG10 in *P. japonica*. Final target sequences obtained by the comparison with these two Coleoptera species were used to identify the corresponding genomic sequence in *A. lythri* (PRJNA947484; Rohlfing et al., 2023) by Blast searches.

Furthermore, the marker VSSC (gene coding for the voltage‐sensitive sodium channel protein) was previously identified as X‐linked marker in the beetle *Leptinotarsa decemlineata* and was used in a CNV study to identify the genetic sex in these beetles (Hawthorne, 2001; Sedláková et al., 2022). Using the tblastx algorithm (Altschul et al., 1990) with the query Ldvssc (XM_023167295.1), the transcript was annotated in the *A. lythri* reference transcriptome and in the corresponding genomic sequence as described above. This annotated contig was additionally used as “sex‐linked” candidate for the qPCR primer design (see below).

All these potential X‐linked genomic contigs served as the basis to design primers with primer3 (version 4.1.0; Untergasser et al., 2012). Primers with a length of 20–25 nt and a melting temperature of about 60°C were to amplify a region of 150–200 bp during polymerase chain reaction (PCR) or quantitative PCR (qPCR), respectively. In total, primers were designed for three X‐linked loci (VSSC, SL4108, SL3442) and one autosomal marker (AL40057) (Table S1 can be found in Appendix S1) and then blasted against the *A. lythri* de novo transcriptome to confirm their specificity.

#### 2.2.3 qPCR experiments and analysis

The DNA of 8 male and 11 female adult beetles and 12 larvae (7 females, 5 males) of HT2 was used to validate the potential x‐markers from the in silico analyses with qPCR. Each qPCR run was performed on a StepOne device (Thermo Fisher Scientific) on a 96‐well plate with three x‐marker and one autosomal marker per DNA sample and at least one female sample as reference on each plate. The DNA concentration of the samples of a plate was measured with the Qubit dsDNA‐BR kit (Thermo Fisher Scientific) directly before the experiment and adjusted to 5 ng/μL. Reactions were conducted in triplicates using the EvaGreen Mastermix (Jena Bioscience, Dortmund, Germany) with 5 ng DNA in each reaction and 200 nM (VSSC, SL4108, SL3442) or 250 nM (AL40057) primer with a three‐step amplification cycle (40 cycles: 98°C for 15 s, 60°C for 20 s, and 72°C for 30 s, detection at last step). Primer efficiency was measured using a standard curve with a serial dilution of DNA from 50 ng/μL to 0.5 pg/μL in steps of 1:10. Specificity of the amplifications was evaluated by a melting curve analysis and separation on agarose gel electrophoresis. Negative controls for each primer without DNA were included on each plate.

The qPCR data analysis was performed in R (version 2023.09.1 + 494). First, all qPCR plates were imported and filtered by the following criteria: (1) Outliers with Ct‐value above 26.5 are discarded, (2) standard deviation of the Ct values of one target gene and sample should be less than or equal to 0.5, otherwise the triplicates differ too much from each other to be reliable, (3) for each target gene and each sample, at least two of the technical triplicates must have a Ct value fulfilling the filter criteria so far, otherwise the target gene of this sample is discarded, and (4) all samples for which no CT value could be calculated for the autosomal gene are discarded because this prevents further analysis.

The 2^−ddCT^ method (Rao et al., 2013) was used to analyze the data. This calculation was performed on each plate individually according to the following steps. First, dCT was calculated as the difference between the Ct value of the autosomal marker and the X‐linked marker for each sample. Hereafter, ddCT was calculated as the difference between the dCT and the dCT of the female reference sample (always HT2) that was run on each plate. Finally, the 2^−ddCt^ was calculated, where the 2 is replaced by the amplification factor of the respective primer of the marker. The amplification factors of the different primers were calculated according to their efficiencies. For further analyses, the results of the individual plates were combined in one table. In females, the expected ratio is around 1, as the same number of gene copies is expected for sex chromosomes and autosomes. In males, the copy number is only half as high because they have only one X chromosome. The ratio to the autosomal marker therefore is expected to be around 0.5.

Samples were assigned a deduced genetic sex according to these expectations. Individuals with values below 0.7 were assigned as males, those with values above 0.8 were assigned as females, and individuals with values between 0.7 and 0.8 were declared unspecified. Biological samples in which sex could not be assigned (unspecified for all sex‐linked markers) were filtered out. The script used also has the option of removing samples for which no clear assignment of sex is possible, that is, if the assignments of the individual sex‐linked markers do not match. To ensure that the determined genetic sex of each sample converged on the same outcome, samples with a standard deviation above 0.3 for the sex‐linked genes were removed at this point. Results were finally visualized in boxplots.

Chi^2^ tests were performed for the number of filtered samples in HT1 plus HT1* compared to HT2 to check that the filtering steps did not cause bias in the data. The specificity and sensitivity for assigning genetic sex to an individual were calculated. R code for filtering, analyzing, and statistics is available in the Data S1.

#### 2.2.4 Implementation of the x‐markers to test for feminization in HT1/HT1*

After evaluating the reliability of the genetic sexing procedure with the HT2 individuals, it was applied to test whether genetic males are hidden among the phenotypic females of HT1/1*. For this purpose, 12 and 16 phenotypic females of HT1 and HT1* adult beetles, respectively, were tested. The phenotype of the beetles was determined morphologically by their internal sex organs or by the female‐specific function of laying fertile eggs.

To determine the genotype ratio within HT1 families, the progeny of 6 HT1 females (2–11 siblings/female), 48 samples in total, was genotyped. The whole larvae were used for DNA extraction with the innuPREP Forensic Kit (Analytic Jena, Jena, Germany) and subsequently for qPCR to determine the genotype. Due to the small size of the larvae, the sample quantity was not sufficient to determine the phenotypic sex by an additional RNA extraction and evaluation of the size and number of dsx splice variants. Due to the exclusive occurrence of females in HT1, these 48 samples were set as phenotypic females in this analysis.

## 3 RESULTS

### 3.1 Read quality/transcriptome assembly

Processing of the reference assembly build with HT1 female and HT2 male reads after redundancy reduction resulted in a set of 32,721 transcripts, with high‐set BUSCO scores (S: 94.8%, D: 1.7%). This supports transcriptome completeness and overall good quality and justifies its use as a reference.

The quality of the reads for the SEX‐DETector analyses was checked by mapping them to the reference transcriptome. The total percentage of mapped RNA reads was approximately 95%, but only about 40% (Figure S1) of the father's reads could be mapped to the transcriptome. As indicated by the sample's GC content (Figure S2), the father's reads differed from the rest, which strongly indicated contamination with prokaryotes, which have a generally higher GC content. The poor quality of the father's RNA is probably due to degradation of the RNA before extraction and the father was probably no longer fully alive at that time. All quality parameters can be found in the Appendix S1.

### 3.2 SEX‐DETector analyses

**FIGURE 1:**
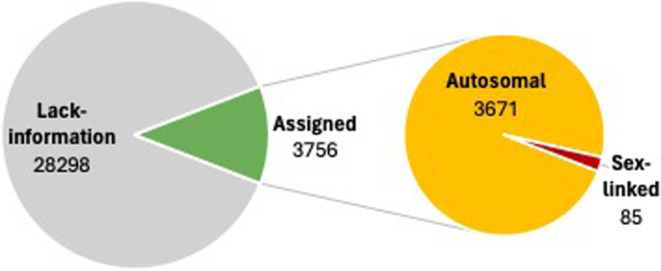
Proportions of assigned transcripts. The percentage of transcripts that could be assigned to a category is shown in green, while autosomal transcripts are shown in yellow and sex‐linked transcripts are shown in red.

The SEX‐DETector analysis was conducted using 14,912 SNPs identified through processing the 12 RNAseq datasets as described above. It revealed that of the 32,721 reference transcripts, 11.7% (3756) of all transcripts could be assigned to either the autosomal or sex‐linked category. The relatively low number of assigned transcripts is presumably due to the poor RNA quality of the father. However, despite this limitation, 85 contigs were identified as sex linked, while 3671 were identified as autosomal linked (Figure 1). From these, two sex‐linked contigs (TRINITY_DN3442_c1_g1 and TRINITY_DN4108_c0_g1) and one autosomal‐linked contig (TRINITY_DN40057_c0_g1) were selected as representatives for evaluating them as sex markers (SL3442, SL4108, and AL40057) in the laboratory using qPCR. In addition to the X‐markers found by the SEX‐DETector approach, the already known X‐marker VSSC in *Leptinotarsa decemlineata* was annotated in the reference transcriptome on the contig TRINITY_30993_c0_g1 and also tested as a potential X‐linked marker by qPCR.

### 3.3 Evaluation of X‐linked markers using qPCR in normal reproducing HT2 beetles and larvae

**FIGURE 2:**
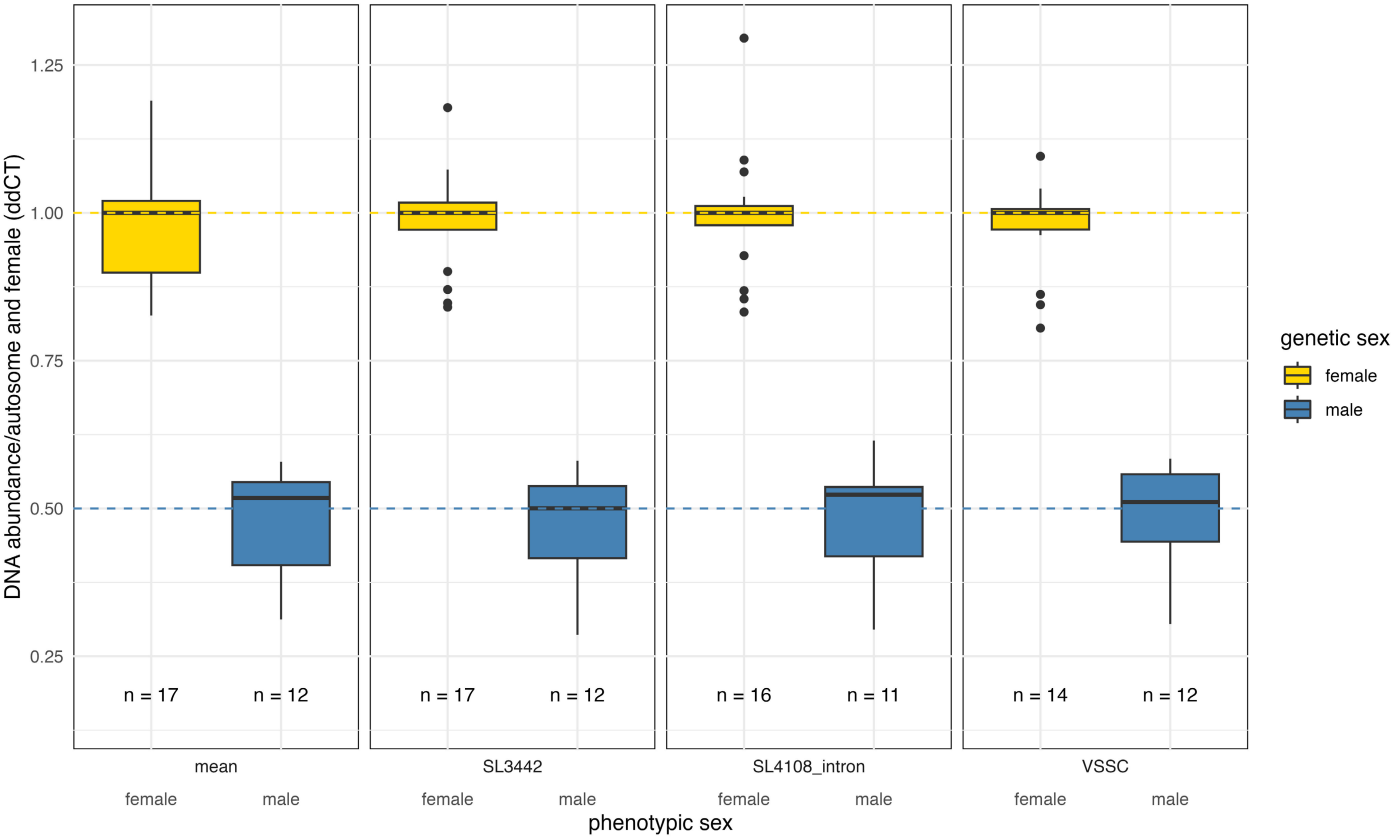
Validation of sex‐linked markers with HT2 samples. Average fold change of three sex‐linked markers (SL3442, SL4108, and VSSC) relative to the autosomal marker (AL40057) and relative to an HT2 female reference sample indicated by the 2^−ddCt^ qPCR analysis method. First plot shows the mean fold change of the three sex‐linked markers. Only HT2 beetles and larvae were tested. Dashed lines represent the expected fold change of male (0.5) and female (1.0) genotypes. Genetic sex is assigned by the fold change and color and phenotypic sex is displayed on the X‐axes. All three markers show the expected fold change in male and female HT2 individuals and are suitable for detecting genetic sex. Box plots give median, first and third quartiles, and outliers; n indicates the sample size.

Potential X‐linked markers established in silico were evaluated using normally reproducing *A. lythri* of HT2. To determine if the X‐linked markers are suitable for identifying larval genotypes, tests were conducted on both developmental stages (larvae and adults). Equal amounts of extracted DNA were used to assess the copy number variation (CNV) of the potential X‐linked and autosomal markers compared to females. The phenotypes of the beetles and larvae were determined before analysis. After data filtering, 17 female and 12 male samples were evaluated concerning their genetic sex. Only one sample of a male larva did not pass the entire filtering process. Ct values of all sample runs are available in Table S2. The potential autosomal locus on contig 40,057 was set to 1 in females and males due to the same expected relative DNA amount of an autosome in both sexes. The potential X‐linked loci VSSC, SL4108, and SL3442 have different DNA abundances in males and females, with fold change in males approximately half that in females. The average fold change of VSSC is 0.97 ± 0.08, of SL3442 0.99 ± 0.08, and for SL4108 1.0 ± 0.11 in females compared to 0.49 ± 0.09, 0.47 ± 0.1, and 0.48 ± 0.1 (VSSC, SL3442, SL4108) in males relative to the autosomal marker (Figure 2). Fold changes in all samples are available in Table S3. The relative DNA quantity, given by the mean of the X‐linked markers, is 0.47 ± 0.1 in males, which is half of that in females (0.99 ± 0.09) (Figure 2), thus corroborating that the tested loci are localized on the X chromosome. In summary, the use of these three sex‐linked markers in combination with the autosomal marker is suitable for detecting the genetic sex of male and female *A. lythri* at different developmental stages.

### 3.4 Genotype assessment of phenotypically exclusively female HT1/1*

The validated X‐linked markers were used to assess the genotype of the phenotypically exclusively female HT1 and HT1*. Sixty phenotypic females of HT1 (12 adult beetles and 48 larvae) and 21 phenotypic females of HT1* (15 adult beetles and 6 larvae) were genotyped. In HT1/1* genotyping, like in HT2, the fold changes were computed based on the Ct values of the sex‐linked markers relative to the autosomal marker and compared to an HT2 female as reference, given the absence of abnormalities in their sexual reproduction. Hence, we can confidently ascertain that this reference sample indeed represents a genetic female with two X chromosomes. Forty‐eight samples of HT1 and 20 samples of HT1* could be evaluated for their genotype after filtering analysis of the results. This implies that 1 of 15 adult samples of HT1* and 9 of 48 larvae samples did not pass the filtering procedure. None of the adult HT1 samples had to be excluded. The χ^2^ test comparing the failed samples between HT2 and HT1/1* indicates no significant difference between the two groups (X‐squared = 0.040667, df = 1, *p*‐value = .8402), suggesting that the filtering process did not introduce bias into the data.

**FIGURE 3:**
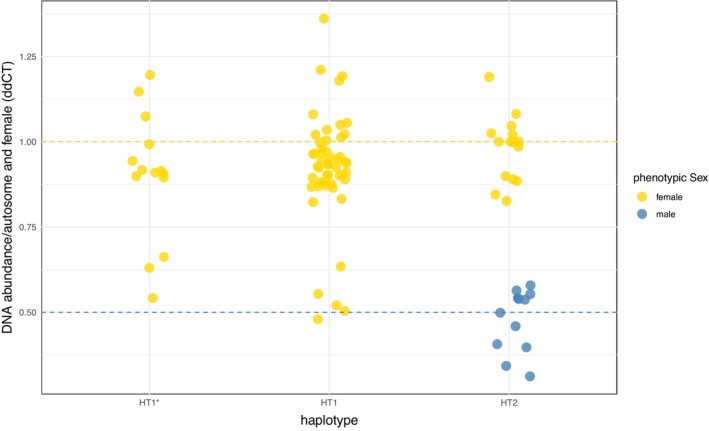
Genetic sex of HT1*, HT1, and HT2 samples. Fold changes of three sex‐linked markers (SL3442, SL4108, and VSSC) relative to the autosomal marker (AL40057) and relative to an HT2 female reference sample revealed by 2^−ddCt^ method in qPCR. HT1*, HT1, and HT2 beetles and larvae were tested and displayed. Dashed lines represent the expected fold change of male (0.5) and female (1.0) genotypes. Genetic sex is assigned by the fold change, displayed on the X‐axes, and phenotypic sex is displayed by color. Note that HT1* and HT1 are phenotypically exclusively female, whereas for HT2, genetic and phenotypic sex are concordant.

The genotyping revealed evidence for two different groups of genotypes in both haplotypes. The average fold change in HT1 samples is either 0.97 ± 0.12 or 0.53 ± 0.06. In HT1* samples, the average fold change is either 0.97 ± 0.11 or 0.61 ± 0.07 (Figure 3). These results indicate that although the HT1/1* samples are all phenotypically female, two different genotypes, having either two X chromosomes (XX) or a single X (XY or X0), are present among HT1 and HT1* individuals. Morphologically, there are no recognizable differences between the genetically different HT1 females. Two of 11 samples of HT1 (18.2%) and 3 of 14 samples of HT1* (21.4%) showed evidence of a single X in their genotypes. To calculate the percentage of single X genotypes in HT1/1*, only adult samples were used because a female with XX genotype can only produce XX offspring which would lead to an underrepresentation of single X individuals.

### 3.5 Genotype ratio in progeny of HT1 families

**FIGURE 4:**
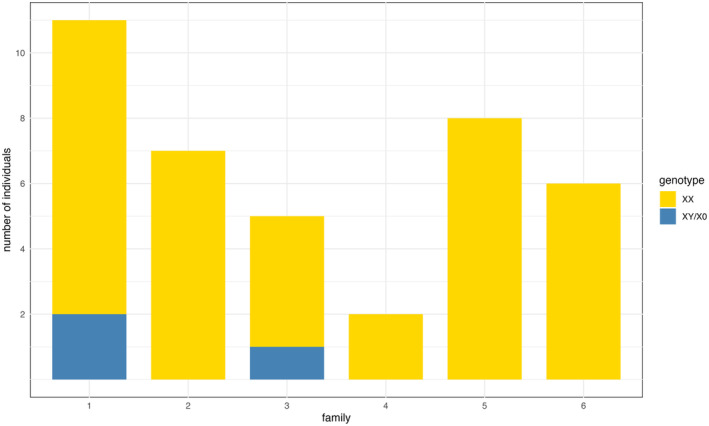
Distribution of genotypes within HT1 families. Genotype composition of siblings of six different HT1 beetles/families was evaluated using the sex‐linked markers in qPCR. Genetic females (XX) are displayed in yellow, and genetic males (XY/X0) in blue.

To investigate the processes at meiosis in HT1, resulting in the development of single X and XX females, we examined the ratio of the single X genotype in the progeny from HT1 families using our newly established qPCR method. Although the siblings were known to stem from the same female, the mother could no longer be included in the analysis. The genotypes of the progeny from 6 HT1 families, each with 2–11 offspring, were determined using all three X‐linked loci. Four families exclusively produced XX offspring, indicating that the mother likely also had an XX genotype, or that the single X genotype was missed due to the small sample size (Figure 4). In two families, both genotypes were present among the offspring, suggesting that the mother had a single X genotype (Figure 4). Within these two families of single X females, the ratio of X:XX was 0.22 and 0.25, respectively. These percentages align with those determined from the adult samples.

## 4 DISCUSSION

While whole‐genome sequencing has become more affordable and accessible in recent years due to advances in sequencing technologies, the processing and analysis of resulting data still represent a significant effort. Assignment of sex chromosomes by whole‐genome sequencing thus remains time and work intensive, and faster methods to identify X‐ or Y‐chromosomal markers are preferable. In this study, we used the SEX‐DETector strategy (Muyle et al., 2016), which infers sex‐chromosome‐linked sequences from more easily accessible transcriptomes based on segregation analyses of a single set of parents and offspring.

We started by generating a family of *A. lythri*, including a mother, father, and 10 offspring (4 females and 6 males), and sequenced their transcriptomes. Using this dataset, we successfully generated X‐linked markers utilizing SEX‐DETector. Despite the suboptimal quality of the RNA and resulting sequences from the father, potential X‐linked markers could still be identified. This underscores the robustness of the SEX‐DETector approach. Furthermore, an independent confirmation of our results was provided through the very recent completion of a chromosome‐level assembly for *A. lythri* by the Darwin ToL initiative (Darwin Tree of Life Project, 2022). All X‐linked markers we identified are located on the putative X chromosome inferred by the Darwin ToL pipeline.

Subsequently, we validated the markers as X‐linked using normally reproducing HT2 beetles. This confirmed that with the selected X‐linked markers (VSSC, SL3442, and SL4108) and the autosomal marker (AL40057), genetic sex assignment is possible. The strict filtering during analysis and the use of three different X‐linked markers avoided misassignments. This discarded only few samples where sex assignment was ambiguous, and the qPCR likely needed to be repeated. However, greater emphasis was placed on achieving 100% specificity albeit with some compromise in sensitivity (around 90%). No bias was generated between the different haplotypes due to the filtering steps. In HT2 beetles, normal sexual reproduction with a 50% distribution of genetic males and females could be confirmed, as reported in previous studies (Jäckel, 2011; Jäckel et al., 2013; Rohlfing et al., 2023). In these beetles, our study corroborates that the phenotype consistently corresponds to the expected genotype, with females having two X chromosomes and males having a single X chromosome, as previously reported for *A. lythri* by karyological analyses (females XX, males XY) (Segarra & Petitpierre, 1982).

In HT1 and HT1*, a strikingly different picture came to light. As documented in previous studies, beetles with these haplotypes exclusively produce phenotypic females (Jäckel, 2011; Jäckel et al., 2013; Rohlfing et al., 2023). Applying X‐linked markers to the phenotypic females of HT1 reveals side by side the presence of females with two X chromosomes and females with a single X chromosome. Nevertheless, these animals are diploid, as evidenced by the consistent Ct values of the autosomal marker. The lack of Y‐specific markers precludes a final decision of whether these females are genotypical XY or X0.

The most primitive karyotype of the Alticinae is supposed to be 11 + Xyp (Smith & Virkki, 1978), where the ‘p’ refers to parachute, indicating a structure (nucleolar proteins that aggregate in metaphase I in the cleft between the X and the tiny Y chromosomes) facilitating proper segregation of the sex chromosomes, despite their unequal size precluding recombination and crossing over points (Dutrillaux & Dutrillaux, 2017; Segarra & Petitpierre, 1982). However, *A. lythri* possesses sex chromosomes of relatively equal size (X and Y) and is classified as karyotype 11 + X + y, with both sex chromosomes remaining unpaired during meiosis I as is also the case in other *Altica* species (Segarra & Petitpierre, 1982, 1990). In almost half of all families of Coleoptera, species exhibit the genotype X0, with the Y chromosome lost independently multiple times within the Polyphaga subgroup (Blackmon & Demuth, 2014; Dutrillaux & Dutrillaux, 2017), although the overall percentage of X0 males in Coleoptera ranges only between 8 and 16% (Dutrillaux & Dutrillaux, 2017; Petitpierre et al., 1988). It is thus not excluded that the Y chromosome might have been lost in the feminized HT1 *A. lythri* males. In several *Drosophila* species, both XY and X0 males have been shown to coexist within a single species (Brown et al., 2020; Voelker & Kojima, 1971).

The possibility of a mixture of different sex chromosomal genotypes underlying the phenotypically female HT1/1* beetles can now also be corroborated by alternative techniques. Paternity studies with *A. lythri* using ddRAD markers support that the HT1/1* females reproduce by gynogenesis, needing copulation only to trigger the development of the egg and that even virgin females lay diploid eggs (Sanken, 2023). In our view, the most likely explanation for this and our current observations is a form of automictic meiosis where two of the female meiosis products fuse in HT1/HT1* individuals to yield a viable zygote (Mirzaghaderi & Hörandl, 2016). This can explain the production of siblings representing both genotypes (XX and XY/X0), as observed in our present study. The meiotic process must entail chromosome and chromatid separation and fusion of the meiosis products while the possibility of apomixis is precluded. Yet, the underlying processes of meiosis still need to be elucidated further. At present, karyological comparisons of the different haplotypes and meiosis stages, including the validation of X‐linked markers via fluorescence in situ hybridization (FISH) are underway (Rohlfing et al., unpublished).

The feminization of genetic males can best explain the XX and XY/X0 we detected in HT1 females. Feminization can be caused by environmental perturbations or stochastic effects but it can also be caused by endosymbiont bacteria, for example, *Wolbachia pipientis* and *Cardinium hertigii* (Kageyama et al., 2012). However, despite the correlation with a specific *Wolbachia* strain in HT1 females, this study does not formally demonstrate that *Wolbachia* is the cause of the sex ratio distortion of the haplotypes HT1/HT1*. To investigate this further, antibiotic experiments to cure HT1 beetles from *Wolbachia* infections are currently underway. So far, in insects, feminization has only been described in Hemiptera (Asgharian et al., 2014; Negri et al., 2006) and Lepidoptera (Hiroki et al., 2004; Narita et al., 2007, 2011; Sugimoto & Ishikawa, 2012), but not in Coleoptera. Although the detailed processes at meiosis leading to XX versus XY or X0 offspring in HT1/1* individuals still need to be elucidated, our study is the first to provide evidence for feminization of genetic males in a beetle species.

## AUTHOR CONTRIBUTIONS

**Kim Rohlfing:** Conceptualization (equal); data curation (equal); formal analysis (equal); investigation (equal); methodology (equal); project administration (equal); software (equal); validation (equal); visualization (equal); writing – original draft (lead). **Malte Grewoldt:** Data curation (equal); formal analysis (equal); investigation (equal); methodology (equal); software (equal); visualization (supporting); writing – review and editing (supporting). **Mathilde Cordellier:** Conceptualization (supporting); formal analysis (supporting); methodology (supporting); project administration (supporting); software (supporting); writing – review and editing (supporting). **Susanne Dobler:** Conceptualization (supporting); funding acquisition (lead); project administration (supporting); resources (lead); supervision (supporting); writing – review and editing (equal).

## CONFLICT OF INTEREST STATEMENT

The authors declare no competing interests.

## Supporting information


Appendix S1.

Table S2.

Table S3.

Data S1.

## Data Availability

The data that support the findings of this study are available in GenBank Bioproject PRJNA1136704, including the data of the reference transcriptome and the raw reads of the 12 transcriptome samples from the SEX‐DETector study. Furthermore, both the raw data and final data from the qPCR approach, as well as the R code used for analysis, are provided in the Data S1.
