# Supplementary material for: Evidence for feminized genetic males in a flea beetle using newly identified X‐linked markers
Source: Ecol Evol. 2024 Aug 12;14(8):e70123. doi: 10.1002/ece3.70123 (PMC11318108; doi:10.1002/ece3.70123)
Supplement: Supplementary file 1 — Appendix S1. [file ECE3-14-e70123-s001.docx]

**Table S1: Oligonucleotides used for CNV analyses in qPCR.**

| Contig /Gene name | Primer-label | 5’-3’ sequence |
| --- | --- | --- |
| VSSC | VSSC_intron_qPCR_f | TTGGTGTTAAGTGTTGCCAAGG |
|  | VSSC_intron_qPCR_r | TTGCCTTCGTTGTACCTATGGG |
| SL4108 | SL4108_qPCR_Intr_f | AGCTACTGTTTCGTCGCTTTCC |
|  | SL4108_qPCR_Intr_r | AAGACGCTGCCCTAAGAAACAC |
| SL3442 | SL3442_qPCR_f | AGTACAATCCAGCCTTGCCC |
|  | SL3442_qPCR_r | GCGAGCAGTGGATCTCTACC |
| AL40057neu | AL_40057_neu_for | ACAATTGAAGTATCAAAATCATTATTTTGC |
|  | AL_40057_neu_rev | ACAGCTATTTTGTTCTAATTTCTTTGTTT |

**Data S1: R code for qPCR analyses. RMD file**


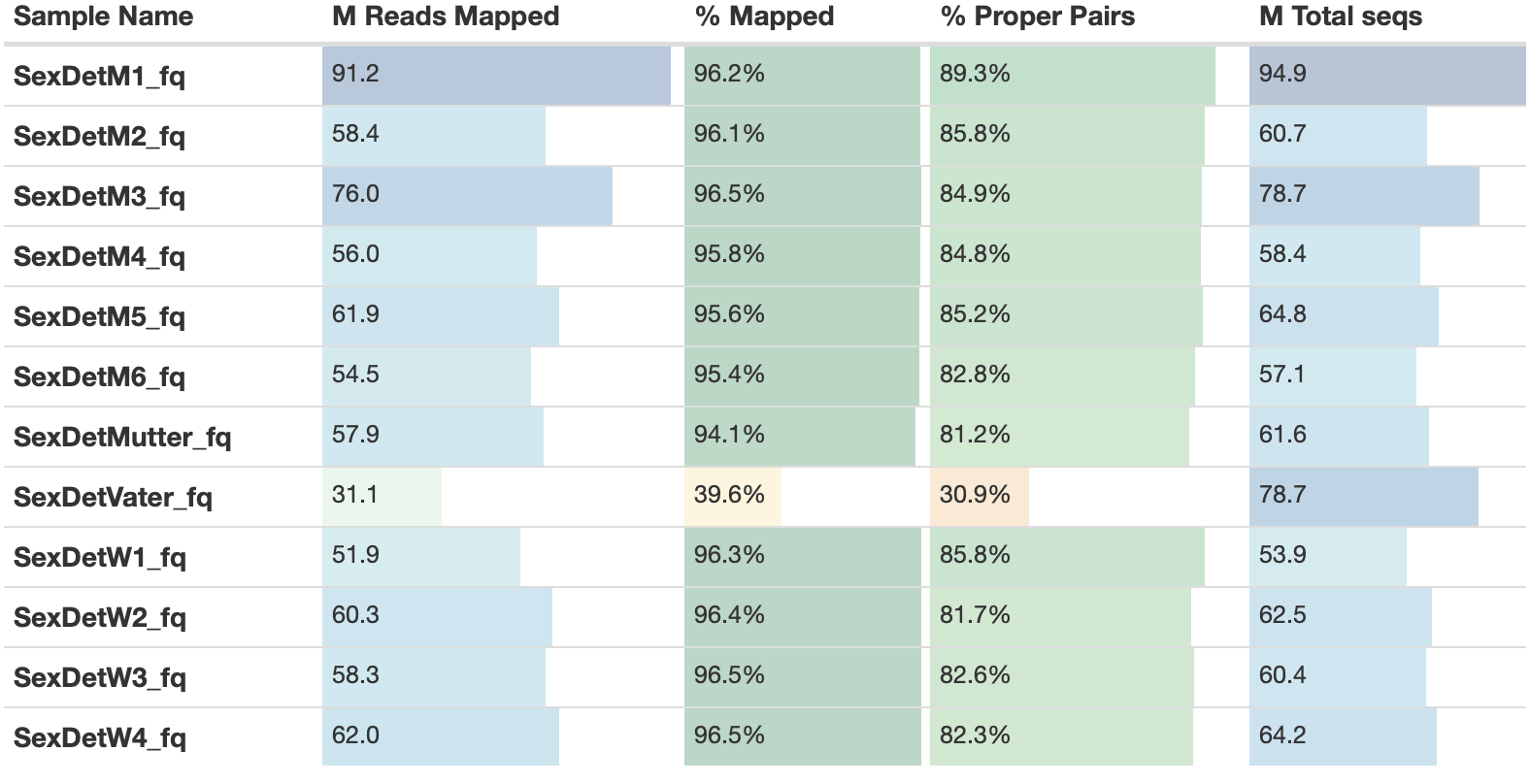


**Figure S1. Proportions of mapped reads.** Trimmed RNA reads from all twelve individuals were mapped to the reference transcriptome.


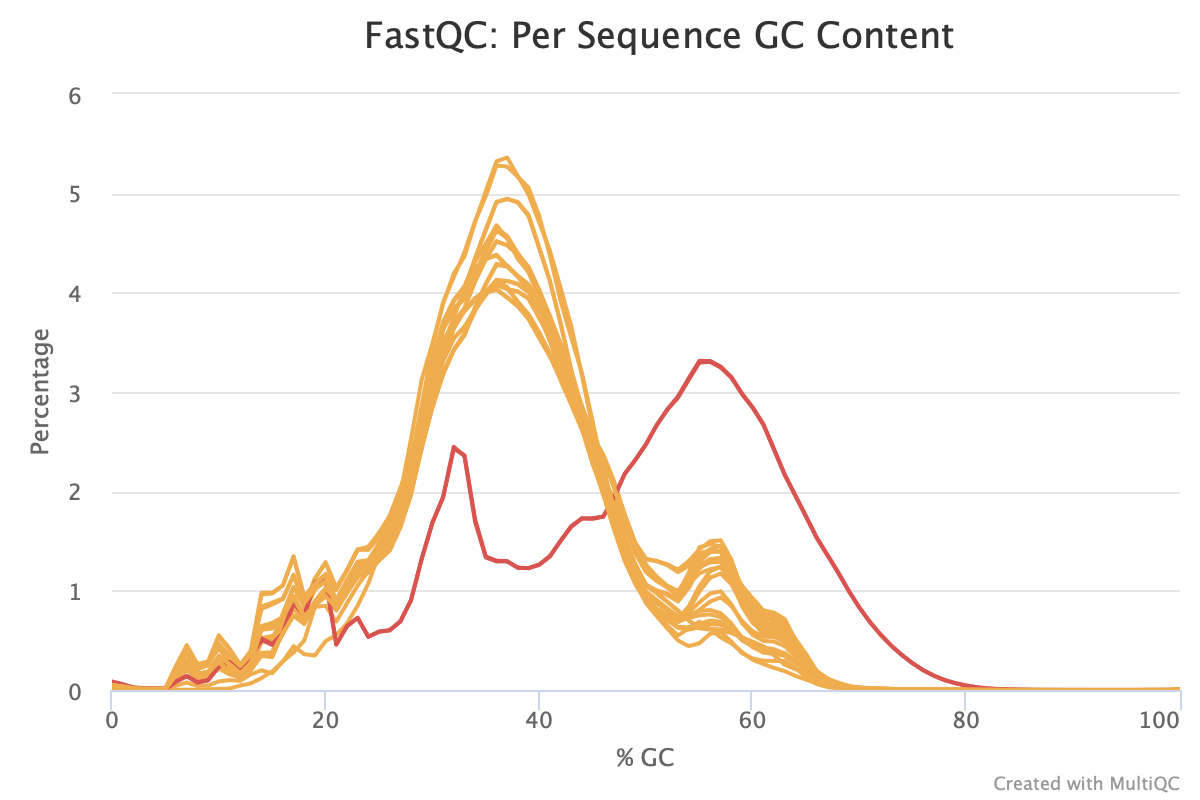


**Figure S2. Per Sequence GC Content.** Distribution of average GC content in each sample. The sample of the father is shown in red, whereas all other samples are shown in orange.

**Table S2. Ct values of all individual qPCR experiments. Excel file**

**Table S3. Fold changes of all biological replicates. Excel file**
